# Estimating the costs associated with the implementation of a best practice model of care for recurrent miscarriage clinics in Ireland: a cost analysis

**DOI:** 10.12688/hrbopenres.13625.1

**Published:** 2022-11-16

**Authors:** Caragh Flannery, Lee-Ann Burke, Paddy Gillespie, Keelin O'Donoghue

**Affiliations:** 1 Pregnancy Loss Research Group, Infant Research Centre, Cork University Maternity Hospital, University College Cork, Cork, Cork, Ireland; 2 Department of Economics, Cork University Business School, University College Cork, Cork, Cork, Ireland; 3 Health Economics and Policy Analysis Centre (HEPAC), Institute for Lifecourse & Society (ILAS),CURAM, SFI Research Centre for Medical Devices, National University of Ireland, Galway, Ireland, Galway, Galway, Israel

**Keywords:** Recurrent miscarriage, recurrent pregnancy loss, cost analysis, health service research, quality improvement, cost estimates, micro-costing, recurrent miscarriage clinics

## Abstract

**Background**

Recurrent miscarriage (RM) affects 1%–5% of the reproductive age population. Given increasing calls for dedicated recurrent miscarriage clinics (RMC), decision makers will require data on the resultant budgetary implications. The aim of this study was to identify the potential costs to the Irish healthcare system of implementing a best practice RMC model of care.

**Methods**

A ‘best practice’ RMC was developed as part of the RE:CURRENT Project. A micro-costing approach was employed by identifying, measuring, and valuing resource usage by unit costs for the RMC for ≥2 consecutive losses. Per patient costs were estimated using two care pathway scenarios: typical and complex. Per patient costs were extrapolated, using population data and published prevalence rates for RM, to estimate the total cost to the Irish health system. A sensitivity analysis was also performed.

**Results**

The cost for a RM patient who has another pregnancy after receiving investigations, treatment and reassurance scans ranges between €1,634 (typical) and €4,818 (complex). For a RM patient who does not conceive again, costs range from €1,384 (typical) to €4,318 (complex). Using population estimates for ≥2 losses, the total cost to the Irish health service ranges from €20,336,229 (complex) to €61,927,630 (typical) for those who progress to pregnancy, and from €7,789,437 (complex) to €22,480,630 (typical) for those who do not progress to another pregnancy. Together, the total cost of the proposed best practice RMC is €112,533,926 with an average cost per patient €1,871.>

**Conclusions**

This study advocates for a new model of care for RMCs in Ireland and provides a set of cost estimates at the patient and healthcare system level. While future studies should explicitly consider the cost effectiveness of this or similar models of care, this analysis provides a valuable first step in providing a detailed breakdown of the associated costs and budget implications.

## Introduction

A miscarriage is the spontaneous loss of a clinically established intrauterine pregnancy before the fetus has reached viability at 24 weeks of gestation ^1^. Recurrent miscarriage (RM) affects 1%-5% of the reproductive age population ^2, 3^. Based on 2017 guidelines from the European Society of Human Reproduction and Embryology (ESHRE), recurrent pregnancy loss is now defined as the loss of two or more pregnancies (previously three or more) ^2^, with the sequence of the miscarriages not necessarily consecutive ^4, 5^. However, it has been estimated that 6% of women have two or more consecutive miscarriages ^2, 3, 6^.

RMs can be considered a multifactorial condition at the population level and in the individual couple ^7^. Studies have identified several causative factors linked with RM, including epidemiological factors such as maternal age and number of previous miscarriages, genetic factors such as parental chromosomal arrangements and embryonic chromosomal abnormalities, and anatomical factors including congenital uterine malformations ^8^. In addition, causative factors include diabetes, thyroid disease, polycystic ovary syndrome, and inherited thrombophilia factors, among others ^9– 11^ Furthermore, chronic endometritis, luteal phase deficiency and high sperm DNA fragmentation levels have also been examined ^2, 3, 10^. Almost 70% of women with two miscarriages will conceive a subsequent pregnancy, with a 70% success rate ^1, 12– 15^. However, a previous live birth does not prevent a woman from experiencing RM, with the risk of further miscarriage reaching approximately 40% after three consecutive pregnancy losses ^13, 14^.

Maternity units across Ireland and the United Kingdom have established Early Pregnancy Assessment Units (EPAUs) to allow for specialised assessment and treatment of women in early pregnancy ^16^. These units focus on the diagnosis and management of miscarriage, providing counselling and support, improving the quality of antenatal care for women with complications in early pregnancy ^17, 18^. Several non-randomised studies suggest that attending a dedicated early pregnancy clinic with psychological support has a beneficial effect, although the mechanism is unclear ^19, 20^. Despite EPAUs, it is recognised that complete care for recurrent pregnancy loss is best offered in a dedicated recurrent miscarriage clinic (RMC) using evidence-based guidelines ^21, 22^. RMCs are consultant-led, non-acute and offer specialist investigations, support, and treatment to women/couples with RM ^21, 22^. They provide care plans to reduce the risk of further losses through treatments and modifiable risk factors ^21, 22^. Currently, there is limited evidence that this approach improves pregnancy outcomes, but some guidelines advocate for this approach in current practice ^15, 23^. Further, RMCs can shorten ‘the interval’ between referral and initiation of treatment with women/couples valuing these care plans for pregnancy after RM ^21^.

Despite clinical practice guidelines for RM, adherence to these guidelines does not guarantee a successful outcome, nor does it necessarily establish a standard of care ^2, 24^. While women/couples with RM are provided with care based on the definition of RM some women/couples are not always treated following the guidelines, resulting in unnecessary tests and costs ^15, 22, 25^. Furthermore, patients can have a strong will to perform diagnostic tests and start treatment ^26^ despite a lack of evidence ^22^. While some evidence-based treatments have improved the outcomes for women/couples with RMs, almost half of the cases remain unexplained ^13, 15^. Many investigations and treatments for RM are controversial; identifying risk factors and effective interventions to prevent miscarriage has become a priority ^13, 27, 28^.

In Ireland, miscarriage occurs in up to 20% of pregnancies, equating to approximately 14,000 miscarriages annually ^18, 29^. However, this is not exact as the number of miscarriages is not officially recorded in Ireland and is most likely underestimated as not all women will attend hospital for miscarriage ^30^. While data from all public maternity hospitals/units in Ireland from 2005 to 2016 identified 50,538 hospitalisations for early miscarriage up to 14 weeks gestation ^31^, hospitalisations numbers have since fallen attributable to outpatient department care in EPAUs ^30^. Following the misdiagnosis of miscarriages cases in 2010, the Health Service Executive (HSE) provided resources to improve the management and staffing of EPAUs across Ireland ^32^. In 2017, the National Women and Infants Health Programme (NWIHP) was established to standardise practices and create more consistent and equitable care across maternity services ^33^. More recently, resources were made available with the setup of the Women’s Taskforce 2019 by the Department of Health to improve women’s health outcomes and their experiences of healthcare ^34^.

In 2016, a review of all 19 maternity hospitals/units found four dedicated RMCs operating ^35^, with six operating across the country in 2021 ^36^. Nonetheless, there exists a lack of clarity, both over what may constitute the best practice model of care, and the feasibility and economic impact of such a model of care in practice. Furthermore, there is uncertainty around how to organise RM care, including what investigations and treatments should be provided ^23, 37^. While RM guidelines exist, clinical practice is inconsistent and poorly organised ^23, 37^.

To this end, the RE:CURRENT (REcurrent miscarriage: evaluating CURRENT services) Project sought to identify, prioritise and seek consensus on a suite of guideline-based key performance indicators (KPIs) for RM services in Ireland. The RE:CURRENT Project ^38^ comprised of several interrelated work packages to evaluate the services provided to those who experience RM to optimise and standardise care in Ireland. These work packages involved the identification, synthesis and appraisal of clinical guidelines (CPGs) through a systematic review of the literature, a qualitative study of stakeholder views on RM services ^39^, the development of guideline-based KPIs ^40^ and an evaluation of RM services. Furthermore, key stakeholder perspectives were involved in the RE:CURRENT Project in the form of a Research Advisory Group comprising parent advocates, healthcare professionals, representatives from support and advocacy organisations, and members involved in the governance and management of maternity services.

The systematic review that appraised CPGs for the investigation, management and follow-up of RM found that women/couples with RM should be referred to individual clinicians/multi-disciplinary teams within specialist clinics and/or elsewhere ^37^. Furthermore, in terms of the structure of care, counselling (psychological/emotional) support and informational support were provided to women/couples from the outset ^37^. Several of the CPGs that defined RM referred to two or more losses, which is also reflected in the ESHRE recommendations ^2^. These CPGs were synthesised and prioritised for inclusion in a suite of guideline-based KPIs for RM care in a modified e-Delphi study with members of the RE:CURRENT Research Advisory Group ^40^. The RE:CURRENT qualitative analysis of interviews with healthcare professionals delivering RM care and women/men who had experienced RM found interrelated themes that conceptualised how RM is defined ^39^. Results highlighted the need for a standardised definition of RM, balancing the evidence base with women/couples needs and healthcare resources. Furthermore, participants noted that while the criteria stipulated three consecutive losses in practice, there was a move to two losses as support is required ^41^.

A key consideration for decision makers regarding the feasibility of implementing a new model of RM care based around such KPIs will be its cost and economic impact to the healthcare system. While RM represents a significant burden to women/couples, the setup of RMCs involves substantial resource costs to the healthcare system, in the form of healthcare professionals’ time, consultations, investigations, treatment options and follow-on care such as early reassurance scans for subsequent pregnancies.

This study reports on the potential costs to the Irish healthcare system of implementing a ‘best practice’ model of care for RMCs, the design of which was informed by the RE:CURRENT Project ^38^. Evidence from cost analysis plays an important role in informing the cost-effectiveness of healthcare interventions and ensuring that available healthcare resources are used efficiently as health policymakers plan for future healthcare services.

## Methods

### Study design

A cost analysis was employed to estimate the costs associated with implementing a ‘best practice’ RMC model into the Irish healthcare system. The study sought to employ a micro-costing approach, a range of primary and secondary data sources, and quantitative and qualitative techniques to identify, measure, and value the resources required to implement the proposed model of care. This costing process was conducted in line with the recommendations in Ireland's national guidance for undertaking health economic evaluation ^42^. All costs were calculated in Euro in 2020/2021 prices, using appropriate medical inflation and purchasing power indices as required. The findings from the cost analysis are presented first, in terms of the cost per patient and second, in terms of the total cost to the healthcare system. The following subsections present the methodological approaches adopted in the analysis.

### Ethical considerations

As this study did not involve the direct collection of patient-level data or any direct interactions with patients and instead used data extracted from publicly available sources and data elicited from expert healthcare professionals to generate the estimates presented in the paper, ethical approval was not required for the cost component of the RE:CURRENT Project.

**Figure 1.**
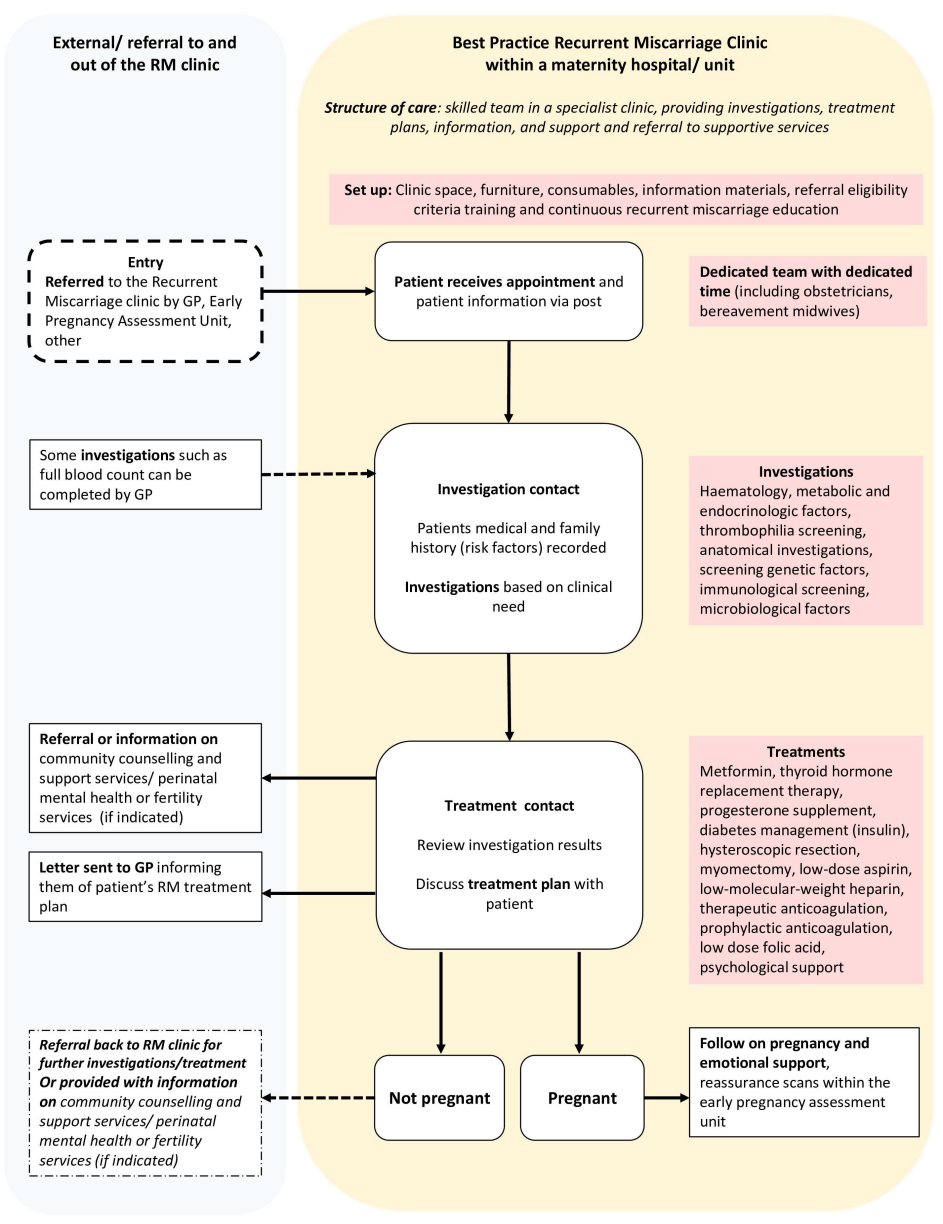
Proposed ‘best practice’ Model of Care for RMC based on KPIs.

Based on findings from the RE:CURRENT Project ^37, 40^ and input from an expert elicitation exercise described below, a flow diagram of a best practice RMC was generated (see Figure 1). In short, a RMC is a consultant-led clinic providing dedicated and focused services to women/couples who have experienced at least two consecutive miscarriages. Women/couples that experience two or more losses, regardless of age are referred to the clinic, usually by the EPAU or general practitioner (GP) (based on eligibility criteria). Women/couples move through the pathway having contacts with healthcare professionals (obstetrician and/or clinical midwife specialist in bereavement and loss) ranging between 30-60 minutes during their investigations, treatment, and subsequent pregnancy care. During this time, a set of recommended investigations such as blood tests, ultrasounds and genetic testing can be carried out based on the individual’s risk factors/history with subsequent treatment options prescribed. These appointments provide information and emotional/psychological support to women/couples alongside investigations and treatment. The number of contacts with clinical midwife specialists in bereavement and loss is patient-led, with some women/couples receiving additional support as required. Depending on the outcome, women/couples can be referred to other specialist services such as social work, counselling, perinatal mental health, genetic counselling, and fertility.

### Cost analysis

Micro costing techniques were applied ^43^, and the developed costing framework was based on standard methods of identifying, measuring and valuing resources items to estimate total costs ^44^. The cost analysis of a best practice RMC model of care was calculated based on the following components: *1. The initial set up costs of a best practice RMC*, *2. The on-going implementation costs of delivering a best practice RMC*, and *3. The subsequent and related care pathway costs*. Total costs were estimated and presented first, per patient and second, were extrapolated to estimate the impact to the healthcare system for the estimated patient population.

### Selection of sources

Various sources were used to estimate resource use and unit costs, including the concurrent RE:CURRENT Project ^38^, and published sources (Guidelines for the Economic Evaluation of Health Technologies in Ireland 2010; Guidelines for the budget impact analysis of health technologies in Ireland) ^42, 45^, salary scales ^46^, hospital department costs (estimates from department managers), and previous costing analysis ^31, 32^, along with data collected from an expert elicitation exercise producing a comprehensive cost inventory (expert input questions and cost data inventory can be found under *Extended data* ^47^). Data on resource use and unit costs was collected from March – September 2021.

### Data collection

Regarding resource items and usage, a list of the expected process steps and materials required were compiled and discussed with the project team adjusting, adding, or excluding steps and materials to match KPIs ^37^. For resource use, parameter estimates that could not be determined from empirical evidence, expert input was sought from clinicians running RMCs/services. Clinical experts were contacted, and, upon agreement of participation, information on the background and purpose of the study was provided, along with a list of possible questions related to each parameter of interest. CF (a post-doctoral researcher) conducted an informal discussion with clinical experts (n=3) from different hospitals with experience of RMCs, with experts providing estimates for each parameter. These discussions (face-face discussion in maternity hospital unit (n=1); telephone discussions (n=2)) lasting up to 30 minutes each helped define the best practice RMC in terms of the following items: target population, patient management, the clinic pathway, investigations, treatment plan, outcomes, and onward referrals.

With respect to unit costs, setup costs such as room hire, medical supplies, equipment and training were obtained from discussions with a finance manager and operations manager from the South/Southwest Hospital Group (SSWHG). Salaries for healthcare professionals involved included obstetricians, midwives (senior clinical midwife specialists in bereavement and loss) and hospital administrators, which were sourced from the HSE’s consolidated salary scales October 2020 ^46^. In line with guidelines, all salaries were adjusted for pay-related social insurance (11.05%), pension costs (4%) and overheads (25%) in Ireland ^42, 45^. A 39-hour working week was assumed for all individuals, and the cost of their time was calculated as cost per minute. All costs associated with recommended investigations for RM based on the KPIs were sourced from discussions with a principal biochemist in the SSWHG. Treatment costs such as quantity and purchasing price of individual drugs were obtained from a lead pharmacist in the SSWHG. All drug costs included VAT. Costs for subsequent pregnancy care such as reassurance early pregnancy ultrasound scans were included.

### Cost estimations

Each individual element of cost was estimated and summed to generate subtotals for each category of cost, and then again summed to calculate a total cost for the ‘best practice’ RMC model of care over a one-year period. The following approach was adopted to present the findings from the cost analysis. First, per-patient costs were estimated for a typical patient and complex patient. As the type of investigations, treatment prescribed, and the number of contacts with healthcare professionals are determined by patient case and complexity, two patient scenarios (a typical patient versus a complex patient) were used to calculate cost per patient. The final set of scenarios was selected based on guidance from the clinical expertise on the study team. A typical case was based on the type of RM cases most often seen in their clinic, assuming that 10% of women would present with a more complex clinical scenario. To cost the per patient outcomes, two estimates were generated, for those who progress to pregnancy, and those who did not progress to another pregnancy.

Second, to extrapolate the cost results to estimate the total cost to the Irish healthcare care system, population data was collected through the Central Statistics Office 2021 (CSO) Ireland and published prevalence rates for RM ^48^. Data on the prevalence of RM for the Irish population is incomplete. This is also due to how RM is variably defined. The definition ranges from two clinical miscarriages, according to both the American Society for Reproductive Medicine ^49^ and the European Society for Human Reproduction and Embryology ^2^, to three consecutive pregnancy losses as defined by the Royal College of Obstetricians and Gynaecologists, which has recently has been updated introducing a new approach ^6, 10^. This new approach offers women/couples support after one miscarriage, initial investigations after two and a full series of evidence-based investigations after three miscarriages ^6^. Based on the RE:CURRENT Project findings and the growing international consensus, this study uses population numbers for women who may experience two or more losses and be treated within a best practice RMC model of care.

**Figure 2.**
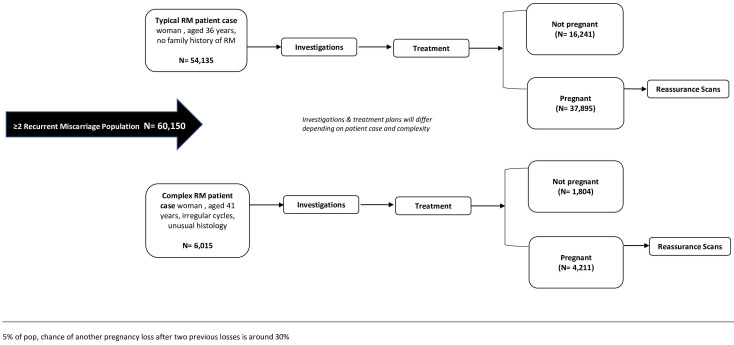
Patient pathway through the recurrent miscarriage clinic with Irish population estimates for two or more losses.

Using estimates from the Central Statistics Office 2021 (CSO) Ireland, the population of Irish women of reproductive age (based on the World Health Organisation definition of reproductive age as 15-49 years) is 1,203,00 ^48^. Approximately 5% of these women will experience at least two consecutive first-trimester pregnancy losses with a 30% chance of another pregnancy loss ^49, 50^. The total cost for the estimated population experiencing two or more losses is presented for a typical patient versus a complex patient ( Figure 2).

### Sensitivity analysis

A sensitivity analysis was performed to explore the uncertainty in the analysis by increasing and decreasing the setup costs, on-going delivery costs, cost per patients (typical/complex patient case) and the population numbers by 10%.

## Results

The results are presented in Table 1– Table 4. The initial set up costs are presented in Table 1 and the on-going implementation costs are presented in Table 2. The total cost estimates by patient are presented in Table 3 and healthcare system estimates are presented in Table 4.

**Table 1.** Total costs to setup a best practice RMC (staffed with one consultant obstetrician and two clinical midwife specialists in bereavement and loss in one maternity unit).

Type of cost	Units	Unit costs	Total Cost
*Set up costs:*			
Consultation room - standard items such as desk, chair, computer, bed, patient chair	2 consultation room	€8,000 fitting out an outpatient consultation room	€16,000
Consumables – equipment, medical supplies such as blood pressure monitor, stethoscope, body weight scales, paper, ink, stationary, needles/syringes, sharp disposal containers, speculums, table covers, gloves, face masks, swabs, urine containers	2 consultation room	€10,000 Standard equipment and supplies included in an outpatient consultation room	€20,000
Materials – development of the information pack for women/couples with RM *	1 pack	€739 health care professionals’ time	€739
*Initial training:*			
Materials – program materials for attendees *	1 pack	€370 healthcare professionals time	€370
Venue – rental	1 classroom	€166 based on room rental in university campus	€166
Trainer – to deliver training	1 trainer × 1 hr session	€46 based on healthcare professionals time	€46
**Total:**			**€37,321**

*Development of information included two bereavement midwives €0.77 per minute Cost data can be found under *Extended data ^47^*

**Table 2.** Total on-going delivery costs for a best RMC (staffed with one consultant obstetrician and two clinical midwife specialists in bereavement and loss in one maternity unit).

Type of cost	Units	Unit costs	Total Cost
*Annual training:*			
Venue – rental	1 classroom	€166 based on room rental in university campus	€166
Trainer – to deliver training	1 trainer x 1 hr session	€46 based on healthcare professionals time	€46
*Clinic space:*			
Venue rental – clinic space renting consultation room	2 consultation rooms x 4 clinics per month per year	€250 per morning/ afternoon session per day	€12,000
**Total:**			**€12,212**

Cost data can be found under *Extended data ^47^*

**Table 3.** Total costs per typical/ complex RM patient for investigations, treatment, and contact with healthcare professionals in a best practice RMC.

Type of cost	Units/ quantity	Unit costs	Typical patient	Complex patient
*Investigations*				
Full blood	1	€20 per test	€20	€20
Thyroid	1	€10 per test	€10	€10
HBA1c (glucose)	1	€10 per test	€10	€10
Day 2-5 hormone	1	€20 per test		€20
TV ultrasound	1	€200 per test	€200	€200
Pelvic MRI	1	€240 per test		€240
ANA test	1	€10 per test	€10	€10
Androgen testing	1	€20 per test	€20	€20
DHEA-S	1	€63.39 per test		€63
APLAS	1	€10 per test	€10	€10
Pregnancy tissues (histology)	1	€193.30 per test	€193	€193
Arry CGH	1	€250 per test	€250	€250
Parental Karyotyping	1	€260 per test	€260	€260
**Subtotal:**			**€983**	**€1,307**
Sensitivity -10% (+10%)			€885 (€1,082)	€1,176 (€1,437)
*Treatment*				
Folate	Both typical & complex 400 mg Treated for 36 weeks	€7.66 30-day supply	€65	€65.11
Aspirin	Typical case 75mg Treated for 33 weeks; Complex case 150mg Treated for 33 weeks	€3.10 28-day supply (75mg); €6.20 28-day supply (150mg)	€26	€51
Progesterone	Typical case 100mg Treated for 10 weeks; Complex case 400mg Treated for 10 weeks	€16.95 30-day supply; €29.00 15-day supply	€39	€136
Low molecular weight heparin	Complex 150mg (syringe) Treated for 34 weeks	€98.20 10-day supply		€2,337
Prednisolone	10mg Treated for 10 weeks	€8.65 10-day supply		€61
**Subtotal:**			**€130**	**€2,651**
Sensitivity -10% (+10%)			€117 (€143)	€2,386 (€2,916)
*HCP time*				
Obstetrician	30 mins per typical consultation, 60 mins per complex consultation	€2.98 per minute	€89	€179
Bereavement midwife	60 mins per consultation *	€0.77 per minute	€46	€46
**Subtotal:**			**€136**	**€225**
Sensitivity -10% (+10%)			€122(€149)	€203 (€248)
*HCP Administrative tasks / * *care coordination time*				
Obstetrician	30 mins per patient	€2.98 per minute	€89	€89
Bereavement midwife	60 mins per patient	€0.77 per minute	€46	€46
**Subtotal:**			**€136**	**€136**
Sensitivity -10% (+10%)			€122 (€149)	122.04 (€149)
*Outcome*				
Pregnant	2 reassurance scans per typical case, up to 4 reassurance scans per complex case	€125.00 per scan	€250	€500
Not pregnant/ another loss *				
**Subtotal:**			**€250**	**€500**
Sensitivity -10% (+10%)			€225 (€275)	€450 (€550)
**Total costs per patient ** **type:**				
Cost of RM clinic based on pregnant outcome *			**€1,634**	**€4,818**
Sensitivity -10% (+10%)			€1,471 (€1,798)	€4,336 (€5,300)
**Cost of RM clinic based on not pregnant/ another loss outcome**			**€1,384**	**€4,318**
Sensitivity -10% (+10%)			€1,246 (€1,384)	€3,886 (€4,750)

*This outcome can result in a full-term pregnancy or another loss. Cost data can be found under *Extended data ^47^*

**Table 4.** Economic impact to the healthcare system for typical/complex RM patient case and pregnant/not pregnant outcomes based on population estimates for Ireland.

	≥2 losses (n=60,150)				
	Typical (n=54,135)		Complex (n=6,015)		
**Outcome**	*Pregnant*	*Not Pregnant*	*Pregnant*	*Not Pregnant*	**Total**
**Pop #**	37,895	16,241	4,221	1,804	
**Cost per** **patient**	€1,634	€1,384	€4,818	€4,318	
**Cost per** **pop**	**€61,927,630**	**€22,480,630**	**€20,336,229**	**€7,789,437**	**€112,533,926**
			*Average cost per patient €1,871*		
**Sensitivity analysis**					
**Pop – (+10%)**	34,106 (41,685)	14,617 (17,865)	3,799 (4,643)	1,624 (1,984)	
**Cost** **– (+10%)**	€55,734,867 (€68,120,393)	€20,232,567 (€24,728,693)	€18,302,606 (€22,369,852)	€7,010,494 (€8,568,381)	
Pop, population					

For 2020/2021, the total cost to set up a best practice RMC model of care was €37,321 (see Table 1). The initial setup costs for one clinic based in one maternity hospital/unit included fitting two out-outpatient consultation rooms with equipment, medical supplies and patient materials and providing initial staff training. Most of these setup costs were required for furnishings (€16,000) and supplies (€20,000).

On-going delivery costs for the RMC include annual training costs for healthcare professionals and clinic space rental. Rental is based on a morning or evening slot, with four RM clinics operating per month (one per week) per year. The yearly total cost is €12,212 ( Table 2).

The total cost of care for a RM patient who goes on to have another pregnancy after receiving investigations, treatment and reassurance scans ranges between €1,634 (typical case) and €4,818 (complex case) ( Table 3). For a RM patient who does not conceive again, costs range from €1,384 (typical case) to €4,318 (complex case).

The annual total cost to the healthcare system estimates were based on data from the CSO in Ireland of 60,150 women who may experience two or more RM. Of these women, 54,135 will be considered a typical case, of which 70% will progress to pregnancy (n=37,895) costing €61,927,630. Thirty per cent of these women will either not get pregnant or experience another loss costing €22,480,630 to the health service. Furthermore, of the 60,150 women, 6,015 will be considered a complex case costing €20,336,229 if they progress to pregnancy (n=4,221) or costing €7,789,437 if they do not get pregnant or experience another loss (n=1,804).

Combining the total costs of a typical and complicated patient case for the estimated two or more RM population, gives a total of €112,533,926 with an average cost per patient €1,871.

### Sensitivity analysis

**Figure 3.**
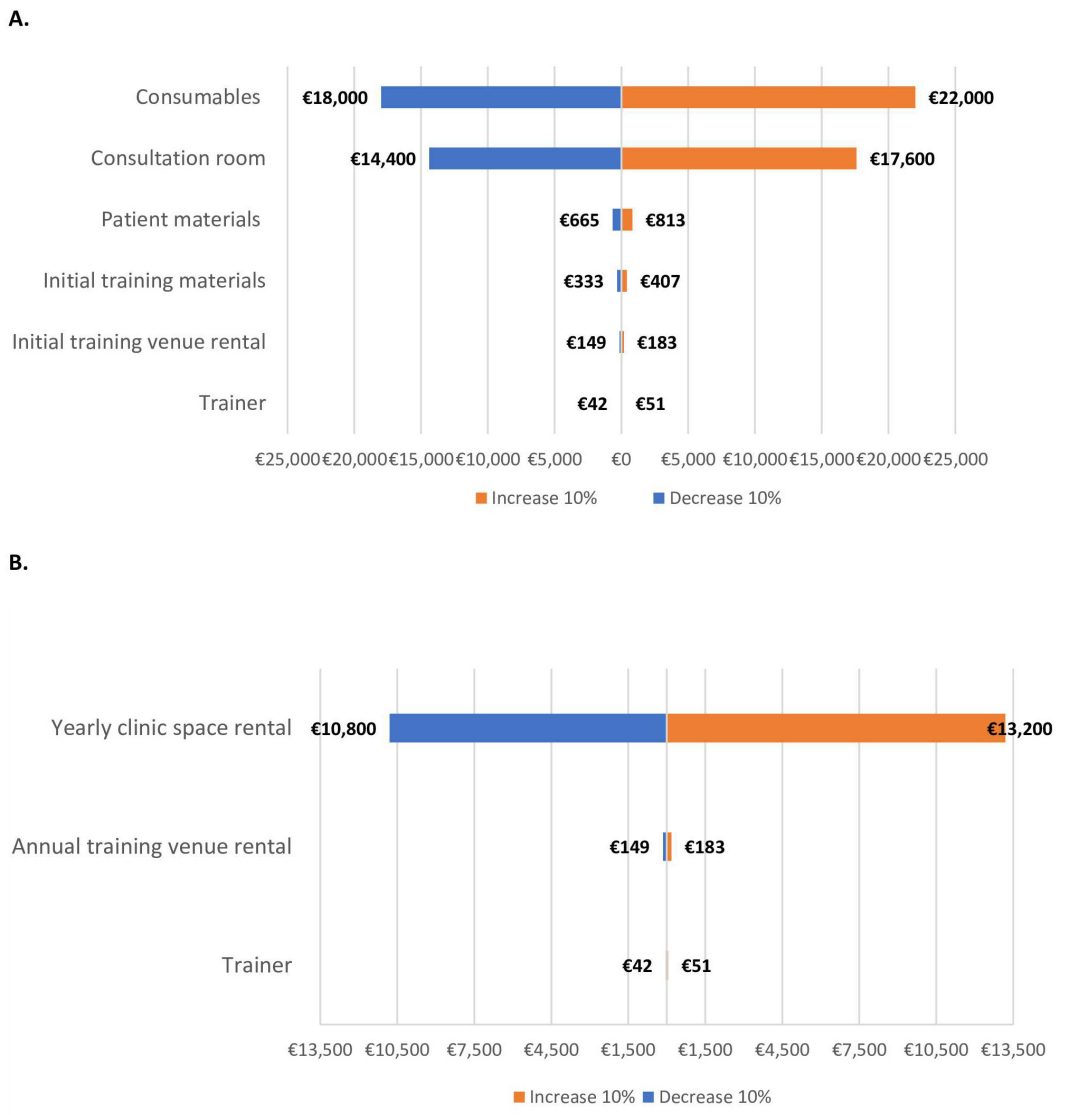
Sensitivity analysis on model costs for set up ( **A**) and on-going delivery ( **B**) of a best practice RMC.

Sensitivity analysis was used to assess how sensitive the results were to fluctuations of 10% in setup and on-going delivery costs, cost per patient and population numbers. The results from the sensitivity analysis in Figure 3A demonstrate that a consultation room and its associated consumables are the main cost drivers for RMC set up. Furthermore, for on-going delivery costs ( Figure 3B), the main cost driver was rental of clinic space (morning or evening slot). For costs per patient, the main cost drivers were investigations and treatments costs ( Table 3). Finally, varying the distribution of the population who may experience a pregnancy after attending a RMC by –(+10%) to 34,4106 (41,685) for a typical case results in total costs of €55,734,867 (€68,120,393) (see Table 4).

## Discussion

Given increasing international calls for a new model of care for RM, this study provides cost and budget estimates relating to a new evidence-based RMC model of care in Ireland. The total cost per patient estimates ranges from €1,634 (typical) to €4,818 (complex) for a pregnancy outcome and €1,384 (typical) to €4,318 (complex) for women who do not progress to another pregnancy. Further, the cost impact to the Irish health service for the estimated population who experience two or more losses ranges from €20,336,229 (complex) to €61,927,630 (typical) for those who progress to pregnancy, and from €7,789,437 (complex) to €22,480,630 (typical) for those who do not progress to another pregnancy. Taken together, the total cost impact of a best practice RMC model of care is €112,533,926 with an average cost per patient €1,871. These estimates will be of interest to policy makers and healthcare decision makers charged with the design, delivery, and financing of care for RMC in Ireland and internationally.

Data on the prevalence of RM for the Irish population is incomplete and the varying RM guidelines of three or more consecutive miscarriage ^6, 10, 49^ will impact the number of women/couples seen/treated and its associated cost impact to the health service. The traditional approach of three or more RMs assumed that the possible causes would significantly differ between the patient groups. However, the research found no such difference, highlighting no justification for denying women/couples investigations and treatment options after two consecutive miscarriages ^21^. Moreover, while definitions and recommendations for RM vary ^37^, there is increasing evidence alongside findings from the RE:CURRENT Project to support offering investigations, information, and emotional/psychological support after two or more RM ^26, 51^. The impact this will have on the health service in terms of resources required to implement a best practice dedicated RMC model of care to cater for this population needs to be considered.

While varying guidelines exist, disjointed RM care leaves women/couples searching for cause and treatment ^23, 37^. The majority of investigations and treatments offered also remain controversial, with lack of consensus amongst health professionals/groups ^28^. While some evidence-based treatments have improved the outcomes for women/couples with RM, other unproven high cost tests and treatments have been marketed to this group ^28^ creating additional economic cost to the health service. This ‘best practice’ model of care for RMC, directly informed by the RE:CURRENT Project will help inform discussions around the evidence and decision-making for the Irish context. Even though almost half the cases of RM remain unexplained ^20^, RMCs are still necessary and valuable. In unexplained cases of RM, supportive care may be all that is recommended ^52^. This is where staff within a dedicated RMC clinic would provide women/couples with continuous support, where they can also be reassured of the cumulative chances of a successful pregnancy (over five years 60–75%) with supportive care alone ^13, 53^.

Undergoing miscarriage is a significant life event, and having RM can magnify the grief experienced following miscarriage ^54^. RM can cause considerable psychological effects, including anxiety and depression, and these emotional symptoms can affect women and their partners in the medium-to-long-term ^2, 55– 57^. The potential adverse emotional and psychological outcomes may impact upon family functioning, relationships, employment, presenteeism, out-of-pocket expenses and further use of health care services ^58– 60^. Furthermore, women who experience multiple RMs may require more frequent contact with healthcare services resulting in additional costs to the health service. A dedicated best practice model of care for RM would offer a specialist service for these women within an understanding and supportive environment. Therefore, investment in staff and training is essential for implementing and delivering a best practice RMC. The impact of RM can have a profound and life-changing impact for the women/couples, and the provision of supportive care should be central to RM management ^54^. Women/couples have previously highlighted the need for more information, psychological support, the inclusion of partners in consultations, and follow-up care ^22, 61^. Therefore, despite the substantial setup, and on-going delivery costs, a dedicated best practice model of care is warranted to provide women/couples the care they need.

In Ireland, access to publicly funded RM care depends on meeting certain referral criteria, often three consecutive miscarriages. Women/couples who do not meet this criterion often pay out of pocket for private care in search of answers for their RM, bearing the financial and emotional costs. Considering these results in the context of the Irish health budget and the recent investment in women’s health and maternity services (NWIHP ^33^, the Women’s Task Force, National Maternity Strategy, Revised Implementation Plan ^36^) economic considerations are particularly important to ensure money is spent efficiently. The estimates provided here can inform and prepare the way for future economic evaluations of this or similar models of care to optimise and standardise RM care. Such analyses could inform future budgetary and resource allocation decisions while maintaining and improving patient outcomes.

### Strengths and limitations

There are limitations to this study. First, a true societal perspective would capture the additional economic impact, such as employment, quality of life and the cost impact on the family. This work is currently underway within the RE:CURRENT Project. While this costing analysis was a useful exercise for budget forecasting and decision making, it does not account for every cost associated with the implementation and on-going delivery of a best practice RMC. Also, this study assumes that maternity hospital/units would be starting from scratch; hence costs reported here are potentially higher. Furthermore, this study collected costs based on one maternity unit with input from two others, and while six RM clinics are operating in Ireland, this study was unable to calculate costs savings, as current practice varies across hospital units. To provide cost estimates per patient, a fixed number of investigations and treatments for both a typical and complex patient type were used based on KPIs ^37, 40^. In a real-world setting, investigations and treatment plans will vary significantly by patient need, history, risk factors, and previous history of loss, directly impacting the costs reported here. This study costs the operation of four RMCs per month over 12 months; however, further clinics may be required depending on population size. Therefore, there is a need for more economic evaluation studies to be conducted on RM and RMCs.

Despite these limitations, this study is the first detailed cost-analysis of a complex care pathway for RM; therefore, the findings can be considered novel. To our knowledge, no recently published studies have disentangled the economic costs associated with the setup, delivery and per-patient costs of implementing a best practice RMC into normal service delivery. This study will enable decision makers to assess in advance what to expect for implementing and delivering a best practice RMC model of care in terms of budget expenditure. Collective findings from this study and the RE:CURRENT Project will inform the standardisation and optimisation of services and support providing a holistic approach to RM care in the Republic of Ireland.

## Conclusions

This study proposes a new model of care for RMC in Ireland and provides a set of cost estimates at the patient and healthcare system level. At present, current provision in Ireland does not appear to meet the needs of the target patient population and alternative models of care, informed by international best practice, should be designed, piloted, and evaluated. In this and in the wider context of increasing constraints on public finances and healthcare resources, evidence on costs and economic impact should also be a key consideration. While future studies should explicitly consider the cost-effectiveness of this or similar models of care, this analysis provides a valuable first step in providing a detailed breakdown of the resources and costs associated with the delivery of RMCs in Ireland.

## Data Availability

OSF: Estimating the costs associated with the implementation of a best practice model of care for recurrent miscarriage clinics in Ireland: a cost analysis. https://doi.org/10.17605/OSF.IO/T48EJ ^47^. This project contains the following underlying data: Investigation_Costs.csv Setup_Costs.csv Training_Staff_Costs.csv Treatment_Costs.csv This project contains the following extended data: Expert Input Questions.docx Data are available under the terms of the Creative Commons Zero "No rights reserved" data waiver (CC0 1.0 Public domain dedication). Conceptualisation & methodology: CF, LAB, PD & KOD formulated the overarching aims and methods of the study. Investigation: CF controlled the data collection and management. Formal analysis: CF conducted the analysis guided by LAB, PD & KOD. Original draft: CF prepared the initial draft. Writing – review & editing: CF, LAB, PD & KOD with all authors approving the final version of the manuscript.
