# Supplementary material for: Estimating the costs associated with the implementation of a best practice model of care for recurrent miscarriage clinics in Ireland: a cost analysis
Source: HRB Open Res. 2022 Nov 16;5:74. [Version 1] doi: 10.12688/hrbopenres.13625.1 (PMC11445642; doi:10.12688/hrbopenres.13625.1)
Supplement: Peer review history

# Reviewer report: Reviewer response for version 1

**DOI:** 10.21956/hrbopenres.14897.r33848

**Published:** 2024-10-01

**Authors:** Justin C. Konje

**Affiliations:** 1 Department of Health Sciences, University of Leicester, Leicester, UK

Recurrent pregnancy loss (recurrent miscarriage) (RPL) remains a major clinical problem that unfortunately continues to defy concerted efforts to identify causes and treatment. Its incidence varies depending on the definition but it is in general accepted that couples who suffer from this problem require care from a dedicated and well informed team. This is the basis for a greater call for specialized clinics for this problem. Such dedicated clinics would provide a structured approach to management that would include evidenced based investigations and targeted treatment including supportive care (which has been shown to be the only treatment that offers benefits to all couples). Development of guidelines help standardize case across countries and indeed the world. What has not been properly investigated/studied is the best model of care for couples with recurrent pregnancy loss and the cost of such care. It is in this context that this manuscript is timely and welcomed. It presents a best practice model of care and more importantly estimated costs of implementing this model of care.

 The team in the RE:CURRENT Project, identified, synthesized and appraised clinical guidelines on the management of RPL and following this process and stakeholder view developed a "Best Model of Care" for RPL and in this study determined the potential cost of implementing this model to the Irish Healthcare System. The cost of care for a couple with RPL who are investigated and treated for their RPL and achieve another pregnancy vary from 1634 Euros(typical) to 4818 Euros (complex). When 2 or more miscarriages was used to identify those with RPL, the total cost to the Irish healthcare system ranged from 20,336,229 to 61,927630 Euros for those who progress to pregnancy and from 7,789,437 to 22,480,630 Euros for those who do not progress to another pregnancy. They concluded that their study advocates for a new model of care for RPL couples in Ireland and provides a set of cost estimates for the patient and the healthcare system.

 This is an important study and should be of interest to clinicians, policy makers as well as managers of various healthcare systems. The attempt to cost treatment should provide a basis for insurance cover for such couples.

 Comments:

1. In the introduction RM is set to affect 1-5% of the reproductive age group but in the introduction this figure rises to 6%.
2. Suggest changing "epidemiological factors" - 4th sentence of the 2nd paragraph (left panel) to "demographic factors".
3. I am uncertain whether the use of "causative factors" in the second a paragraph is strictly speaking correct. These are associated factors with miscarriages and causation has not been proven in most of them. Could this be modified?
4. In the "Data Collection" section it would seem that 3 clinical experts were involved in the generation of the "Best Practice" approach. Is 3 really enough? Should more clinicians not have been involved to improve on the acceptability of generated model? This is a major weakness as the list of investigations costed may reflect this. A large number of those investigations would not be offered in contemporary RPL clinics - at least in the UK and North America.
5. What is the definition of a typical and a complex patient? This is the basis of the cost differential and readers would like some clarity here.
6. The end-point was progress to pregnancy. Was this pregnancy ending in a live birth or just pregnancy?
7. What is the rationale for the annual training cost for healthcare workers?
8. How many ultrasound scans were included in the Best Practice model?
9. What was the basis for the treatment options as well the list of investigations for RPL. The various guidelines including the recent guideline from FIGO do not recommend some of these investigation. Could removing them reduce the total cost?

Is the work clearly and accurately presented and does it cite the current literature?

Yes

If applicable, is the statistical analysis and its interpretation appropriate?

Not applicable

Are all the source data underlying the results available to ensure full reproducibility?

No source data required

Is the study design appropriate and is the work technically sound?

Partly

Are the conclusions drawn adequately supported by the results?

Yes

Are sufficient details of methods and analysis provided to allow replication by others?

Yes

Reviewer Expertise:

Recurrent pregnancy loss. fetal medicine and endometriosis

I confirm that I have read this submission and believe that I have an appropriate level of expertise to confirm that it is of an acceptable scientific standard.

# Reviewer report: Reviewer response for version 1

**DOI:** 10.21956/hrbopenres.14897.r33178

**Published:** 2023-10-17

**Authors:** Peter May

**Affiliations:** 1 Centre for Health Policy and Management, Trinity College Dublin, Dublin, Ireland

Thanks for the opportunity to review this article estimating the costs associated with a best practice model of care for recurrent miscarriage (RM) in Ireland.

 This is an important and interesting topic, understudied in research and underdiscussed in public, and so the overall study of which this is one part is very welcome.

 I found the article very well written and presented. Here are my comments, I hope they're helpful.

**MAJOR COMMENT**

***Estimated cost to the system***

 If I understand Figure 2 right, you are saying that your population of interest is 5% of all women aged 15-49 in Ireland in 2021, since 5% of women will experience RM at some point between the ages of 15-49. This is ~1200000*0.05 = ~60,000. And you then estimate cost per patient and then cost to the Irish health system. 

 At first, I assumed this cost to the system is annual, but this doesn't track. If 5% of 15-49yos will experience RM *at some point* then that means that *in 2021*, 5% of 49yos had already done so, a small minority of 15yos had already done so, and the proportion to have already done so between ages 16 and 48 increases, presumably with an S-shaped distribution. Therefore, and depending on the age distribution within the cohort and the mean age of RM occurrence, the clinical population *in 2021* who had already experienced RM was roughly half of the 5% of women aged 15-49 who will do so at some point in their lives. 

 OK, so your 'cost to the system' is not annual (and you never say that the cost is annual, and in many cases the treatment pathway may be more than a year). But in that case, how do we interpret it? It seems like you are estimating the total cost of RM care for this specific cohort; women aged 15-49 in 2021? In that case though, should you consider other factors? E.g. among those who already had RM, time from occurrence to 2021 will vary widely, being longest in the oldest, and need for the service will vary inversely with that time period as well as being confounded by later potential pregnancies. (Not to say that those who are further from the events and/or since had children are not still affected by RM, but that both intensity and volume of need likely varies). This varying need may also be distributed differently in your typical and complex groups. And the fact that roughly half of the cohort have not yet experienced RM in 2021 but will do so means that their costs need to be estimated for the future? And then the total timeframe of analysis is to 2055 (when the 15-yos in 2021 will be 49)?

 I think the paper would benefit from consideration of these issues and in particular specifying a timeframe for the system-level estimate. Either I have misunderstood and you can clarify the timeframe and the interpretation of your 'cost to the system' estimate. Or you should consider revising the analysis and reporting a bit so that you are either (a) estimating a population-level cost for one year or a few years, or (b) estimating to 2055 the costs for the cohort of women aged 15-49 in 2021.

 My own prejudice would be towards (a) a population-level year by year estimate, which would be easier to calculate, easier to interpret, and more useful for policy and planning.

**MINOR COMMENTS**

***1 Typical and complex patients***

 This dichotomy is central to the analysis and results, including the abstract, but first appears in the manuscript in a roundabout way in the 'Methods>Cost estimations' section. Given its importance, consider putting the the idea in the introduction with relevant references and adding a new subsection 'Methods>Patient pathways' where the typical and the complex are defined in their own right before you start on estimating the associated costs.

***2 'Reproductive age population'***

 I found this phrase (first appearing in the abstract) a bit confusing. RM affects 1-5% of the 'reproductive age population'. I am not familiar with the clinical literature so perhaps this is the standard language. As an outsider I was wondering, men and women both have age spans during which they are reproductive; these spans may not be the same; where there are two partners, both are affected by RM but some non-negligible proportion of pregnancies occur without a partner; once RM has occurred it affects the people involved for some indeterminate and heterogeneous amount of time....

 As such, I wasn't sure who this was really describing, and the phrase appears in a few places. At the same time I acknowledge that it's very complicated to be precise and concise. In main analysis you are using 5% of all women of reproductive age so stating this specifically throughout may be helpful. 

***3 Statistics in the introduction***

 Following on from (2), I found some of the statistics in the Intro hard to parse. I believe absolutely it's a huge problem but understanding the numbers, who the people are, and the timeframe, was harder. 

 E.g. in the opening para you say that 1-5% of the relevant population are affected, and 6% of all women are affected (presumably at any point in the life course?). Intuitively I can't put these numbers together, and if there's such uncertainty in the first shouldn't there also be in the second? In your main analysis you adopt 5%, using different references (refs 49, 50) to those in the first paragraph of the paper (refs 2, 3).

 E.g. you say that there's ~14,000 events annually but this is "likely underestimated". It's already a pretty rough estimate so what are the known and unknown parameters? Not all women attend hospital for miscarriage, but does this 14,000 figure incorporate some estimate of these non-attendances? If no then it's *certainly* a huge underestimate; if yes then wouldn't it be more accurate to say that it's unknown or uncertain than that it's an underestimate (why would an estimate be an underestimate, or why can't you fix that yourselves)?

***4 Affiliations***

 In my version 1 pdf, author affiliation #3 uses the word 'Galway' three times, the word 'Ireland' twice and then places Galway in Israel. This implies a fault in either the submission forms or my geography knowledge.

Is the work clearly and accurately presented and does it cite the current literature?

Yes

If applicable, is the statistical analysis and its interpretation appropriate?

Not applicable

Are all the source data underlying the results available to ensure full reproducibility?

Yes

Is the study design appropriate and is the work technically sound?

Yes

Are the conclusions drawn adequately supported by the results?

Yes

Are sufficient details of methods and analysis provided to allow replication by others?

Yes

Reviewer Expertise:

Health economics

I confirm that I have read this submission and believe that I have an appropriate level of expertise to confirm that it is of an acceptable scientific standard, however I have significant reservations, as outlined above.

# Reviewer report: Reviewer response for version 1

**DOI:** 10.21956/hrbopenres.14897.r35664

**Published:** 2023-09-13

**Authors:** Pankaj Bahuguna

**Affiliations:** 1 University of Glasgow, Glasgow, Scotland, UK

Thanks for the opportunity to review this paper. This paper presents a cost analysis for recurrent miscarriage clinics in Ireland. Undoubtedly, the topic is of great importance given the burden of problem and cost assessment done by authors is good, though I have several concerns regarding the methods and results presented. First, it is not clear whether the cost assessment was done from an economic or financial perspective. Choosing a perspective has some bearing on what input costs we include, how we handle these costs in the analysis and who is our targeted audience (policy makers)? Second, authors should have included the economic burden of ‘recurrent miscarriages’ in the introduction section. If there is no information available in Ireland’s context, authors could have presented any information available in literature for other countries. This will be helpful for readers to understand intensity of problem and need to address it. Third, it is important to know contextually, are there adequate trainers in the system to impart the training to healthcare professionals involved in RMCs? Also, if we are considering universal provision of RM services, are there adequate RMCs/healthcare professionals to meet the demand for ‘recurrent miscarriage care’ in Irish populations? Please see my detailed comments below:

**Introduction**

- In the introduction, no information on economic burden of ‘recurrent miscarriages’ has been included. In case of dearth of such evidence in the Irish context, it would be worth to cite similar evidence from any other country/region. Authors set the context based on burden of the problem and thus, need for cost assessment. Also, they recommend undertaking cost-effectiveness for same as future research. Hence, it becomes more important to reflect the existing evidence on economic side of the problem. Currently, there is only one generic sentence about unnecessary tests and associated costs in introduction.
- Second last para of introduction, “While RM represents a significant burden to women/couples,…………….” What burden authors are trying to highlight here? Is it economic or social or mental or Overall? It would be good to specify.

 

**Methods**

- My main concern in methods is whether the cost assessment has been done from financial or economic perspective? In some places, authors state, these are economic costs, and a micro-costing approach has been employed for this cost analysis but to me, the cost analysis seems to be from a financial perspective. If these are economic costs, there is no hint in the manuscript how capital costs were analysed? Why annualization of capital resources were not done? Life of different capital resources assumed? I assume even trainings has a life more than a year, therefore, should be treated as a capital resource and analysed? When would experts think healthcare professionals would need refresher training? All these considerations are important for economic costing. Many input costs listed in Table 1 fall within the category of capital costs. Such as set-up costs (consultation room material), within consumables BP monitors, weighing scales, stethoscopes, weighing scales, speculums etc.
- Ideally this costing should have been done from both economic and financial perspectives. Financial cost estimates are relevant from payer’s perspective or budgetary perspective, while economic estimates are important for undertaking a full economic evaluation.
- In figure 2, the age given for 2 groups seems to be average age. Please specify this in the footnote for better clarity of readers.
- I couldn’t understand the rationale behind using a variation of ±10% in sensitivity analysis. Varying the population by 10% might be good assumption to represent the uncertainty but 10% for input resource data seems a bit illogical. Ideally, different uncertainty factors should have been used for different costs such as set-up costs, training costs, investigations, treatment etc. Resource inputs should be varied by an uncertainty factor and then costs to be estimated under ‘Sensitivity analysis’ instead of simply varying the estimated costs. For example, number of investigations should vary under sensitivity analysis instead of estimated cost of investigations. I would like to hear more from authors about their justification for assumption used.

**Results**

- Table 2, Do we have enough trainers in the system to impart the trainings or are the trainings planned in a phased manner? Is there a need for ‘training of trainers’ to have enough trainers? If yes, probably including these costs would be relevant.
- Do we have enough healthcare professionals to be trained for RMCs? If not, there might be a need to recruit more healthcare professionals. The cost of recruitment must be different from simply accounting for wage rates. Moreover, costs depend on type of recruitment (regular vs contractual) contingent on benefits provided. All these considerations are important for estimating economic costs.
- Table 3, What do ‘Unit costs’ signify? Are these reference costs? What exactly do these costs include? (Only operational costs or operational+non-operational both?)

**Discussion**

 Some of the discussion points also highlight the need to address points raised in my comments.

- First paragraph, “These estimates will be of interest to policymakers and healthcare decision makers……..”

Type of policymakers stated in this line direct more toward need for financial cost estimates.

- Fourth paragraph, “Therefore, investment in staff and training is essential for implementing and delivering a best practice RMC”

This sentence also highlights my point regarding additional resource requirements (mainly HR) for provisioning of RM services.

- Strengths and Limitations, first para, “further clinics may be required depending upon population size”

This sentence also highlights the need to assess additional resource requirements for universal provisioning of RMCs. Unless we have evidence on scaling up these services at population level, this evidence provides incomplete information to healthcare decision-makers in terms of budgetary requirements for implementation.

Is the work clearly and accurately presented and does it cite the current literature?

Partly

If applicable, is the statistical analysis and its interpretation appropriate?

Yes

Are all the source data underlying the results available to ensure full reproducibility?

Yes

Is the study design appropriate and is the work technically sound?

Partly

Are the conclusions drawn adequately supported by the results?

Partly

Are sufficient details of methods and analysis provided to allow replication by others?

Partly

Reviewer Expertise:

Health Economics

I confirm that I have read this submission and believe that I have an appropriate level of expertise to confirm that it is of an acceptable scientific standard, however I have significant reservations, as outlined above.
